# Supplementary material for: MicroRNA-29a-5p attenuates hemorrhagic transformation and improves outcomes after mechanical reperfusion for acute ischemic stroke
Source: Noncoding RNA Res. 2025 May 28;14:96–106. doi: 10.1016/j.ncrna.2025.05.016 (PMC12173523; doi:10.1016/j.ncrna.2025.05.016)
Supplement: Multimedia component 1 [file mmc1.docx]

**SUPPLEMENTAL MATERIAL**

**Supplementary Table S1.**

**Supplementary Figure S1.**

**Supplementary Figure S2.**

**Supplementary Figure S3.**

**Supplementary Figure S4.**

**Supplementary Table 1.** The top twenty hub genes were calculated by the MCC method in the CytoHubba plugin.

| **Rank** | **Name** | **Score** |
| --- | --- | --- |
| 1 | Ctnnb1 | 4.83×10^7^ |
| 2 | Wnt5a | 4.83×10^7^ |
| 3 | Lrp6 | 4.83×10^7^ |
| 4 | Wnt3 | 4.83×10^7^ |
| 4 | Wnt4 | 4.83×10^7^ |
| 6 | Wnt9b | 4.83×10^7^ |
| 6 | Wnt2b | 4.83×10^7^ |
| 8 | Fzd2 | 4.83×10^7^ |
| 9 | Fzd4 | 4.83×10^7^ |
| 10 | Wnt7b | 4.83×10^7^ |
| 11 | Fzd3 | 4.83×10^7^ |
| 12 | Wnt16 | 4.83×10^7^ |
| 13 | Dvl3 | 4404996 |
| 14 | Fzd1 | 4037046 |
| 15 | Fzd8 | 730800 |
| 16 | Gsk3b | 51120 |
| 17 | Ptpn11 | 8422 |
| 18 | Egf | 8158 |
| 19 | Hgf | 6103 |
| 20 | Kdr | 5524 |

**Supplementary Figure 1.**

**
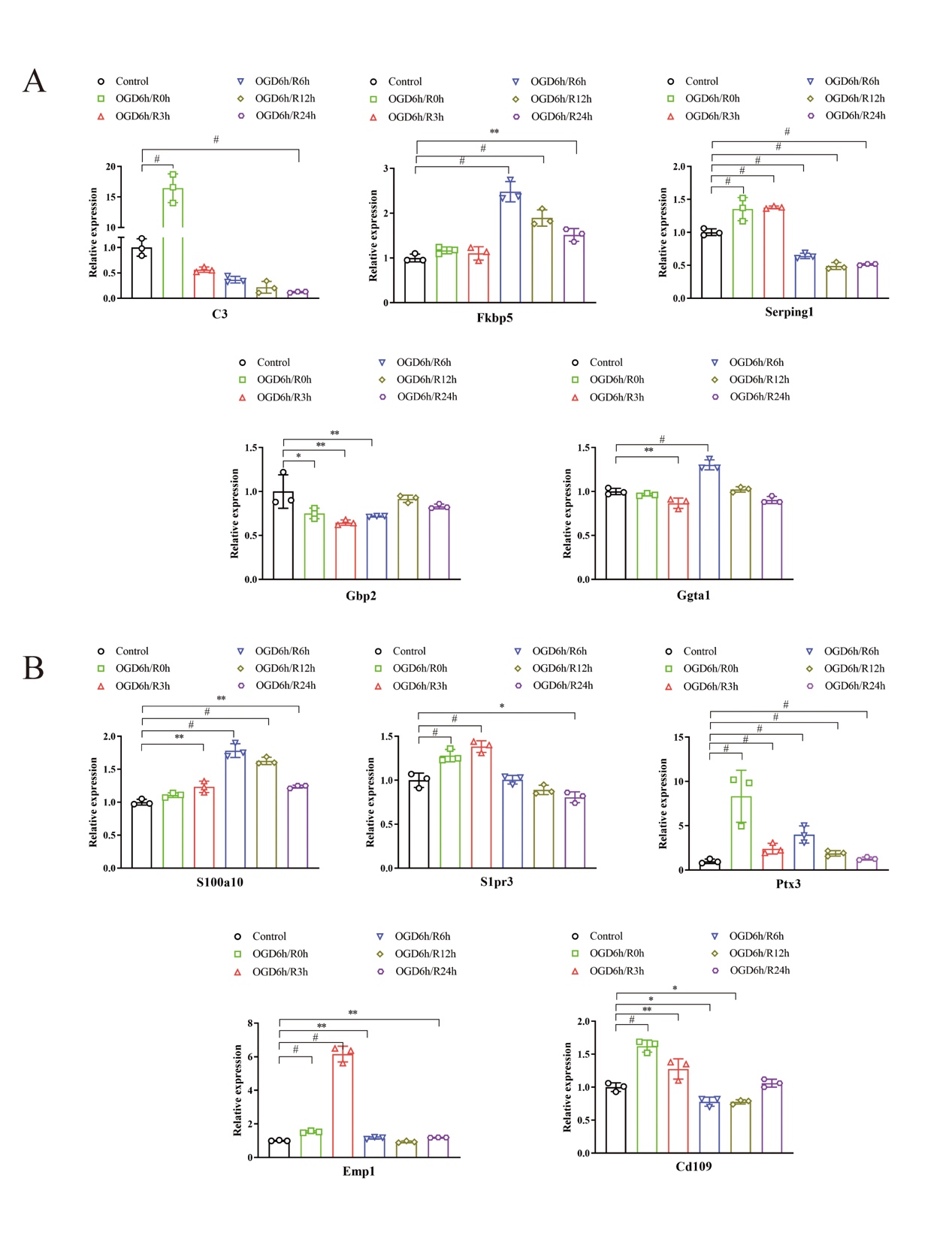
**

**Supplementary Figure 1.** Dynamic expression of A1 and A2 reactive astrocytes markers in astrocytes after OGD/R. In rat astrocytes with oxygen-glucose deprivation for 6 hour and reoxygenation at 0, 3, 6, 12, and 24 hours, the mRNA expression of five A1 reactive astrocytes markers, including C3, Fkbp5, Serping1, Gbp2, and Ggta1 (**A**), and five A2 reactive astrocytes markers, including S100a10, S1pr3, Ptx3, Emp1, and Cd109 (**B**). Each sample was tested in triplicate. *P<0.05; **P<0.01; #P<0.001.


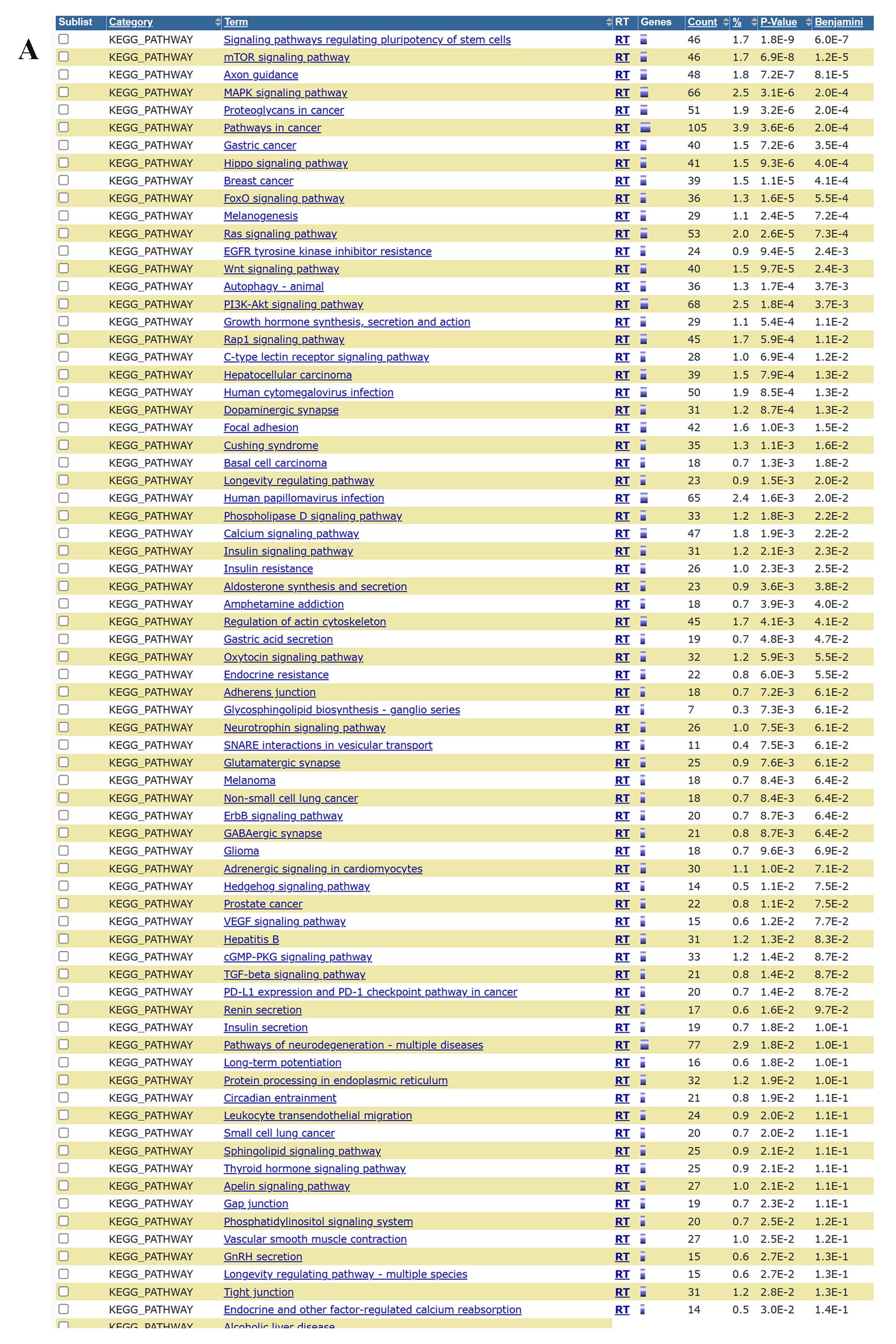
**Supplementary Figure 2.**


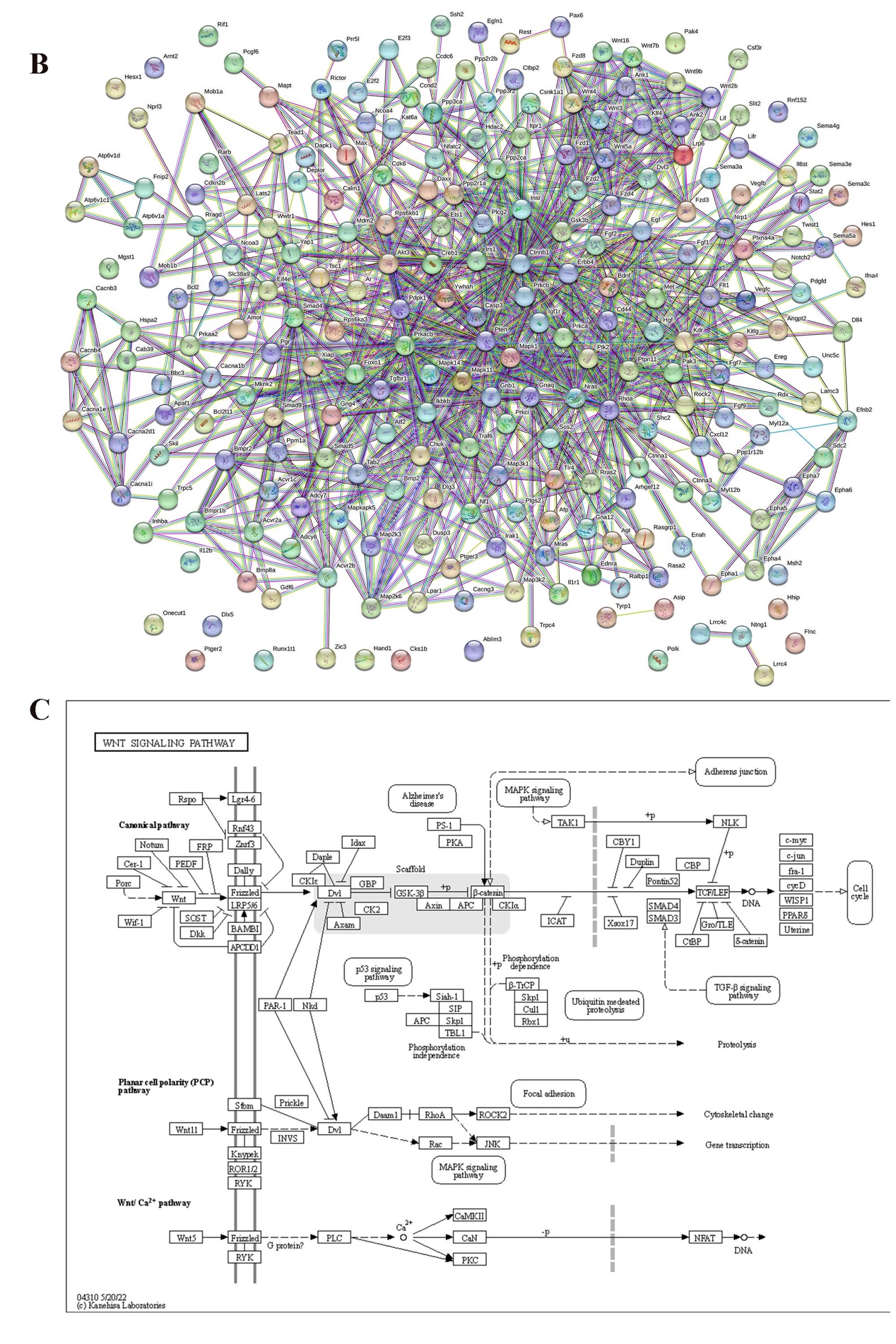


**Supplementary Figure 2.** Bioinformatics predicts target genes for miR-29a-5p. **A,** the enriched KEGG pathways of the predicted target gene by DAVID Bioinformatics Resources. **B,** target genes from the top ten enriched KEGG pathways to construct the protein-protein interaction network by the STRING.

**Supplementary Figure 3.**


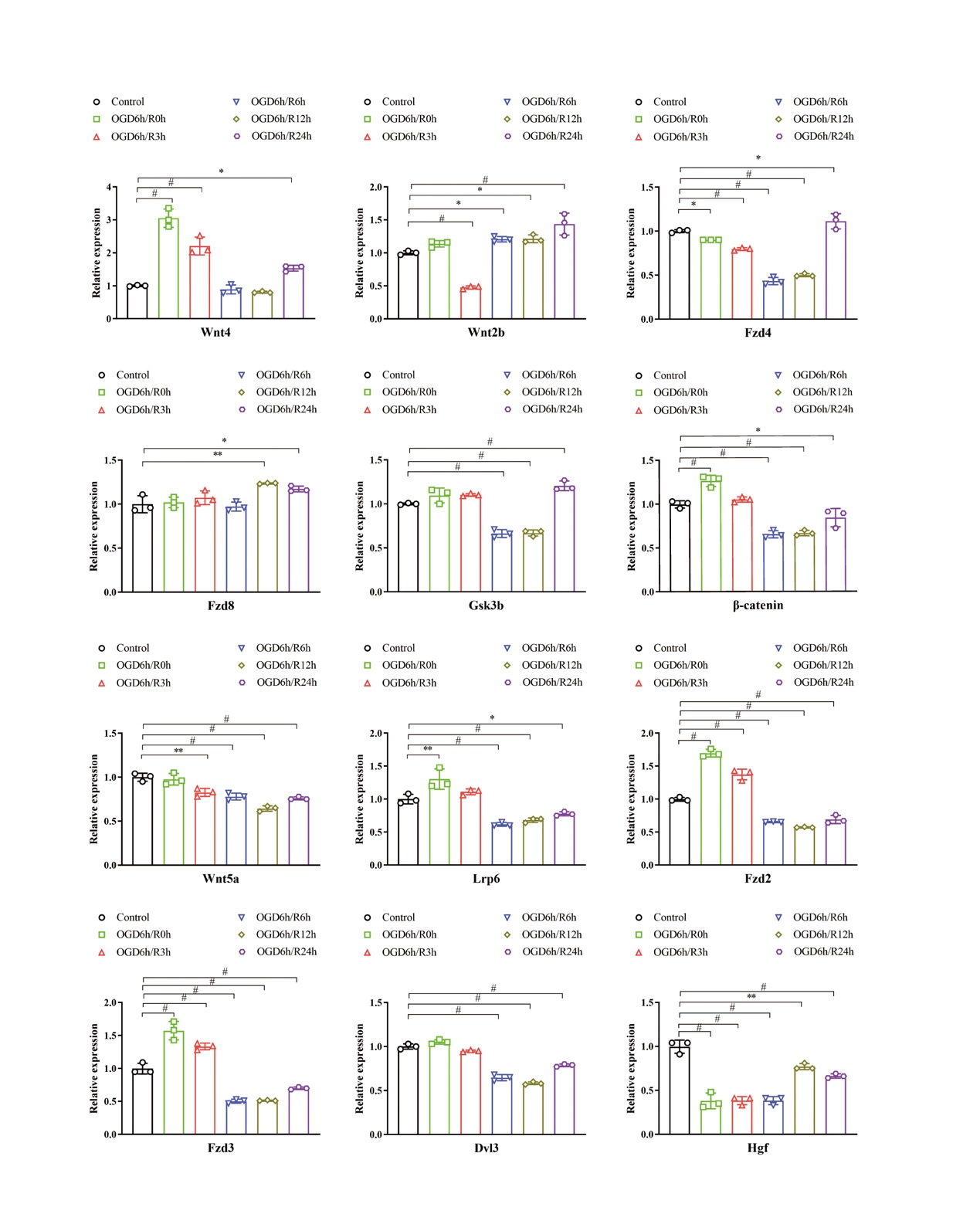


**Supplementary Figure 3.** Twelve hub genes with significantly differential expression in the astrocyte OGD/R model. Twelve hub genes identified by bioinformatics analysis had the significantly differential expression in the astrocyte OGD/R model. Five genes (Wnt4, Wnt2b, Fzd4, Fzd8, Gsk3b) were significantly upregulated, and seven (β-catenin, Wnt5a, Lrp6, Fzd2, Fzd3, Dvl3, Hgf) were downregulated 24 hours after OGD/R. Each sample was tested in triplicate. *P<0.05; **P<0.01; #P<0.001.

**Supplementary Figure 4.**

**
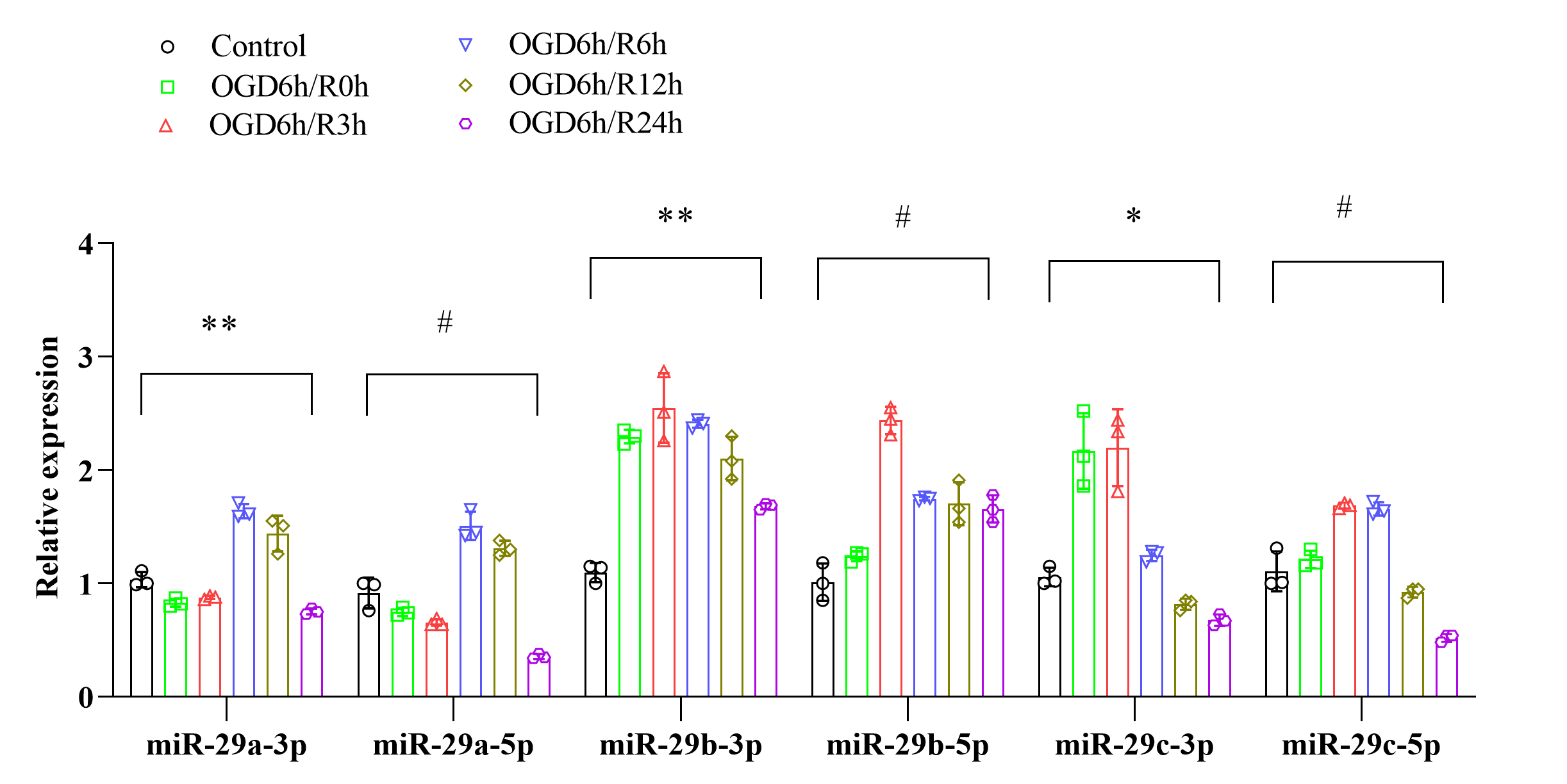
**

**Supplementary Figure 4.** The decreased expression of miR-29a-5p and miR-29a-3p in the astrocyte OGD/R model. There was decreased expression of miR-29a-3p, miR-29a-5p, miR-29c-3p, and miR-29c-5p in OGD/R-treated astrocytes. MiR-29a-5p had the highest decreased expression among the six microRNAs of miR-29 family. Each sample was tested in triplicate. *P<0.05; **P<0.01; #P<0.001.
